# Familial forms of disorders of sex development may be common if infertility is considered a comorbidity

**DOI:** 10.1186/s12887-016-0737-0

**Published:** 2016-11-29

**Authors:** Raja Brauner, Flavia Picard-Dieval, Henri Lottmann, Sébastien Rouget, Joelle Bignon-Topalovic, Anu Bashamboo, Ken McElreavey

**Affiliations:** 1 Fondation Ophtalmologique Adolphe de Rothschild and Université Paris Descartes, Paris, France; 2 Assistance Publique-Hôpitaux de Paris, Hôpital Necker-Enfants Malades, Service de chirurgie viscérale pédiatrique, Paris, France; 3 Human Developmental Genetics, Institut Pasteur, Paris, France

**Keywords:** 46,XY disorders of sex development, Anorchia, Cryptorchidism, DSD, Infertility, Hypospadias, Premature menopause, Premature ovarian insufficiency

## Abstract

**Background:**

Families with 46,XY Disorders of Sex Development (DSD) have been reported, but they are considered to be exceptionally rare, with the exception of the familial forms of disorders affecting androgen synthesis or action. The families of some patients with anorchia may include individuals with 46,XY gonadal dysgenesis. We therefore analysed a large series of patients with 46,XY DSD or anorchia for the occurrence in their family of one of these phenotypes and/or ovarian insufficiency and/or infertility and/or cryptorchidism.

**Methods:**

A retrospective study chart review was performed for 114 patients with 46,XY DSD and 26 patients with 46,XY bilateral anorchia examined at a single institution over a 33 year period.

**Results:**

Of the 140 patients, 25 probands with DSD belonged to 21 families and 7 with anorchia belonged to 7 families. Familial forms represent 22% (25/114) of the 46,XY DSD and 27% (7/26) of the anorchia cases. No case had disorders affecting androgen synthesis or action or 5 α-reductase deficiency. The presenting symptom was genital ambiguity (*n* = 12), hypospadias (*n* = 11) or discordance between 46,XY karyotyping performed in utero to exclude trisomy and female external genitalia (*n* = 2) or anorchia (*n* = 7). Other familial affected individuals presented with DSD and/or premature menopause (4 families) or male infertility (4 families) and/or cryptorchidism. In four families mutations were identified in the genes *SRY*, *NR5A1, GATA4* and *FOG2/ZFPM2*. Surgery discovered dysgerminoma or gonadoblastoma in two cases with gonadal dysgenesis.

**Conclusions:**

This study reveals a surprisingly high frequency of familial forms of 46,XY DSD and anorchia when premature menopause or male factor infertility are included. It also demonstrates the variability of the expression of the phenotype within the families. It highlights the need to the physician to take a full family history including fertility status. This could be important to identify familial cases, understand modes of transmission of the phenotype and eventually understand the genetic factors that are involved.

## Background

The term “disorders of sex development” (DSD) has been defined as “congenital conditions in which the development of chromosomal, gonadal, or anatomical sex is atypical” [1]. DSD constitutes a spectrum of disorders that affect the genito-urinary tract and the endocrine-reproductive system. Anorchia, or embryonic testicular regression (vanishing testis syndrome), is defined as the absence of testes in a 46,XY individual with a male phenotype [2]. It affects one in 20,000 male births [3] and occurs in 1/177 cases of cryptorchidism [4]. Although some patients with anorchia present with genital ambiguity [5] or microphallus [6], most have a normal phenotype. The differentiation of the male genital tract and external genitalia is dependent on anti-Müllerian hormone (AMH) and testosterone, suggesting that functional testes were present but disappeared in utero in these cases. The families of some patients with anorchia may include individuals with 46,XY gonadal dysgenesis. This has led to the hypothesis that anorchia is part of the clinical spectrum of 46,XY gonadal dysgenesis [7]. We and other investigators have observed a variable expression of the clinical phenotypes between family members carrying the same pathogenic mutation (*NR5A1, GATA4* and *FOG2/ZFPM2*) [8–10]. Families with 46,XY DSD have been reported, but they are considered to be exceptionally rare, with the exception of the familial forms of partial or complete androgen insensitivity [11].

We analyzed data obtained from 114 cases of 46,XY DSD and 26 cases of anorchia for the occurrence in their family of one of these phenotypes and/or ovarian insufficiency and/or infertility and/or cryptorchidism. Our data are based on the clinical experience of one pediatric endocrinologist and reveal a surprisingly high frequency of familial forms when premature menopause or male infertility is included.

## Methods

### Patients

A retrospective study chart review was performed for 140 patients evaluated by a senior pediatric endocrinologist (R Brauner) in a university pediatric hospital at a single institution between 1981 and 2014 (33 years). This patient population comprised 114 patients with 46,XY DSD and 26 patients with 46,XY bilateral anorchia. Of these 114 patients, 10 had disorders affecting androgen synthesis (2 patients with 3-β hydroxysteroid dehydrogenase deficiency, 1 patient with *CYP11A1* deficiency and 3 patients with 5α-reductase deficiency) or action (4 patients with partial androgen insensitivity). None of these patients presented with the familial form. This conclusion was based on self-reported family history and on clinical assessment of the sibs of the proband.

Among the 32 familial cases included in the present study, 12 were previously reported as anorchia (*n* = 7) [12], gonadal dysgenesis (*n* = 4) [13] and/or as the first description of the mutation (*n* = 3) [8–10].

### Methods

The Ethical Review Committee (Comité de Protection des Personnes, Ile de France IV) approved this study (IRB n° 00003835). The following parameters were extracted from the medical report: consanguinity, family history relevant to gonadal anomalies, infertility or precocious menopause (arrest of menstruations before 40 years [14]), length and weight at birth, comorbidity or malformations, age and symptoms at first evaluation. Clinical examination included an evaluation of the dimensions of the genital tubercle and the position of the meatus (penoscrotal, midshaft or glandular) and palpation of the labio-scrotal and inguinal areas for the presence and consistency of the gonads. Internal genitalia were evaluated by pelvic ultrasound examination (cases 3–8, 11, 17, 18, 20, and 23), together with magnetic resonance imaging (MRI) in one patient (case 6) because the uterus was not observed. Seven patients underwent genitography (cases 1, 3, 7, 8, 11, 17, and 23). Ultrasound examination was carried out to assess kidney malformations (cases 4, 8, 9–12, 14, 16, 17, 18, and 32).

Gonadal dysgenesis was defined by the presence of Müllerian structures at genitography and/or surgery and/or histological examination. The patients classified as having hypospadias had a normal penis length (greater than 30 mm, −2 SD) [15] and bilaterally or unilaterally palpable inguinal or intrascrotal gonads. Anorchia was defined as the absence of testicular tissue at surgery or by the presence of a small nodule of residual fibrous tissue in a 46,XY individual with a male phenotype.

Nine patients were given testosterone heptylate (3 or 4x100 mg/m2 given IM every 15 days) in the neonatal period before the sex assignment and/or before the genitoplasty.

The endocrine evaluation was performed before surgery. In the patients with DSD, congenital adrenal hyperplasia (normal basal plasma concentrations of 17-OH progesterone in all cases) or insufficiency (normal basal plasma concentrations of adrenocorticotropin hormone and cortisol in cases 3, 8, 10, 11, 13, 14, 17, 20, 22, and 23) or a defect of testosterone biosynthesis were excluded. Partial androgen insensitivity was excluded by the clinical-biological data (low plasma testosterone concentrations during the first months [16] and an increase in genital tubercle after testosterone administration) and by the absence of mutation in exons 1 and 2–8 of the receptor gene in cases 1, 2, 4, 11, 16 and 23–25 (exon 1 not evaluated in case 23). 5α-reductase deficiency was excluded by measuring the testosterone-to-dihydrotestosterone ratio in the basal state (cases 1, 2, 3, 10, 11, 12, 13, and 25) or after stimulation with human chorionic gonadotropin (hCG, cases 6, 7, 17, 20, and 23) and by the absence of mutation in the corresponding gene in cases 11, 16 and 25. Leydig cell function was evaluated by measuring the basal plasma testosterone concentration and after stimulation with hCG (3 or 7×1500 IU, *n* = 9) with samples taken the day after the last injection. The hCG test was not performed on patients with a basal plasma testosterone concentration > 2 ng/mL (except in case 4). Plasma testosterone was measured by using RIA after extraction with Orion reagents (Cis biointernational, Gif-sur-Yvette, France). The sensitivity was 0.07 ng/mL. Plasma hormone concentrations were measured using different immuno-assays during the study period. Each new assay method for a given hormone was always cross-correlated with the previous method to ensure that the results are comparable throughout the entire study period. Plasma concentrations were measured for AMH (except in cases 7, 17, 20, 28 and 32) and inhibin B (except in cases 4, 7, 8, 17, 20, 23, 28, 29 and 32) [17–19]. Plasma AMH was measured using the AMH/Müllerian-inhibiting substance ELISA kit (Immunotech-Beckman, Marseille, France). Plasma inhibin B was measured by ELISA using Oxford Bio-Innovation reagents (Diagnostic Systems Laboratories-France, Cergy-Pontoise, France). Conventional histologic examination of the gonads was performed after gonadectomy (cases 6, 17, and 20) or testicular biopsy (case 25 at 24 years of age).

### Genetic analyses

For each patient, cytogenetic analysis was performed on peripheral blood leukocytes. The chromosome complement was determined by examining 40–50 metaphases from each patient. The *SRY, NR5A1, GATA4, FOG2, INSL3, LGR8 and MAP3K1* genes were sequenced as previously described [20–25] in all patients with DSD until the genetic diagnosis was determined (except in case 24, for which the brother, case 16, had been sequenced). In the patients with anorchia, the complete open reading frames of *SRY, NR5A1, INSL3, MAMLD1* and the T222P variant for *LGR8* were sequenced as previously described [20–25].

## Results

**Fig. 1:**
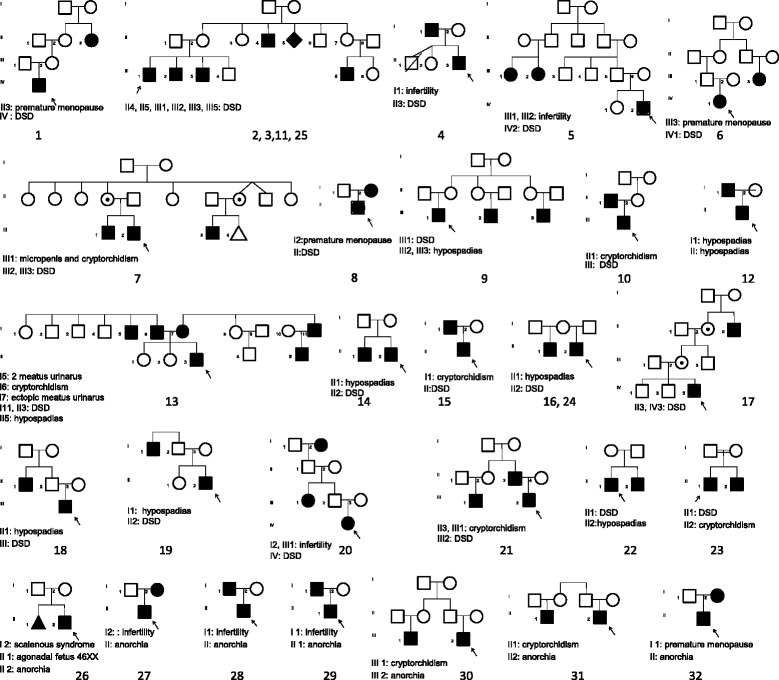
Pedigrees of 25 probands (1 to 25) with DSD belonging to 21 families and 7 probands (26 to 32) with anorchia belonging to 7 families. Squares represent male family members, and circles represent female family members. Symbols containing a black dot represent apparently unaffected carriers of the mutation

Of the 140 patients, 25 probands with DSD belonged to 21 families and 7 with anorchia belonged to 7 families. Familial forms represent 22% (25/114) of the 46,XY DSD and 27% (7/26) of the anorchia cases. The patients with familial form were classified according to the presenting symptom of the index case in two groups: 46,XY DSD with a female phenotype, genital ambiguity or hypospadias (cases 1 to 25) or 46,XY anorchia (cases 26 to 32, Fig. 1).

### 46,XY DSD

Eight patients had comorbidities or somatic anomalies, including two cases with ectodermal dysplasia (cases 6 and 20), one with a congenital cervical mass of 25x20x8 mm containing epitheloid and/or gigantocellular granulomas (case 4), one with bilateral clinodactyly of the 5^th^ finger (case 17), three with retarded prenatal (case 13) or postnatal growth (cases 14 and 22) without defined etiology and one with bilateral vesico-ureteral reflux (case 15).

The gender of rearing was female in 4 patients with female presentation or Prader I to III due to gonadal dysgenesis (cases 5, 6, 17, 20), and male in 21 patients (Table 1). There was no change in the gender after this assignment. The first evaluation was made either prenatally (*n* = 2), before the age of 6 months (*n* = 11), between the age of 6 and 12 months (*n* = 3) or after one year of age (*n* = 9). The presenting symptom was genital ambiguity (*n* = 12), hypospadias (*n* = 11) or discordance between 46,XY karyotyping performed in utero to exclude trisomy and female external genitalia (cases 5 and 6).

**Table 1:** Clinical characteristics of 25 patients with familial DSD

Case	Sex	Age at first evaluation	Presentation	Prader	Localization of the meatus	Genital tubercle (mm)		Testosterone dose (mg)	Gonad	
						before	after		Right	Left
1	M	1 d	genital ambiguity	IV	penoscrotal	15×7	30×12	4×25	s	i
2	M	1 d	genital ambiguity	IV	glandular	20×10		4×50	s	s
3	M	3 d	genital ambiguity	IV	penoscrotal	25×10		4×50	np	np
4	M	3 d	genital ambiguity	IV	penoscrotal	20×8	30×10	4×25	i	i
5^a^	F	3 d	prenatal karyotype	0	normal				np	np
6	F	4 d	prenatal karyotype	I	normal				np	np
7^a^	M	7 d	genital ambiguity	IV	penoscrotal	13	22×15	3×30	i	i
8^a^	M	21 d	genital ambiguity	III	penoscrotal	15×10	30×15	4×40	s	s
9	M	0.1 y	hypospadias	IV	penoscrotal	30×25			s	s
10	M	0.1 y	hypospadias	IV	penoscrotal	30×10			s	s
11	M	0.1 y	genital ambiguity	IV	penoscrotal	20×10	28×10	4×37.5	s	s
12	M	0.3 y	hypospadias	IV	distal	35×15			s	s
13	M	0.4 y	genital ambiguity	IV	midshaft	25×10		3×50	s	s
14	M	0.6 y	hypospadias	IV	penoscrotal	40×15			s	s
15	M	0.8 y	hypospadias	IV	penoscrotal	35×15		3×50	i	i
16	M	0.9 y	hypospadias	IV	midshaft	40×20			s	s
17^a^	F	1.5 y	genital ambiguity	III	penoscrotal	5×5			np	np
18	M	1.8 y	hypospadias	IV	glandular	35×10			i	s
19	M	3 y	hypospadias	V	midshaft	45×15			s	s
20	F	3.5 y	genital ambiguity	II	glandular	18			i	np
21	M	3.9 y	hypospadias	IV	glandular	50×15			i	s
22	M	7.6 y	hypospadias	IV	glandular	45×15			s	s
23	M	10 y	hypospadias	V	penoscrotal	40×12			i	i
24	M	13.6 y	genital ambiguity	IV	penoscrotal	75×35			np	np
25	M	15 y	genital ambiguity	IV	midshaft	65×25			i	i

*DSD* disorders of sex development, *i* inguinal, *s* scrotal, *np* not palpable

^a^Genetic mutations

Cases 2,3,11 are brothers and case 25 is their maternal uncle. Cases 16 and 24 have the same mother (see pedigree)

Among the patients evaluated during the first 6 months of life, 6 patients had basal plasma testosterone concentrations greater than 1 ng/mL and 7 patients had low concentrations of plasma testosterone (Table 2). Of these, two had gonadal dysgenesis (case 5 with a mutation in the *SRY* gene and case 6) and also had low or undetectable concentrations of inhibin B and AMH. One had a mutation in the *GATA4* gene (case 7) and one in *NR5A1* gene (case 8). The 3 other patients with low testosterone concentrations are brothers (cases 2, 3, 11) and they had normal plasma inhibin B and AMH concentrations. Their maternal uncle (case 25) had a normal pubertal increase in testosterone and pubertal development but normal then low inhibin B associated with azoospermia that was confirmed by testicular biopsy. After stimulation with hCG, the testosterone concentration was > 2 ng/mL in 2 of the 7 patients evaluated (cases 4 and 20). The basal plasma follicle stimulating hormone (FSH) concentration was increased (>9 IU/L) in 2 patients (cases 6 and 17 with gonadal dysgenesis) and normal in the others who were in the prepubertal age range, except cases 24 and 25, which were pubertal. The plasma inhibin B concentrations were normal except in the cases with gonadal dysgenesis (cases 5 and 6) and in case 14, which was not reevaluated. The plasma AMH concentrations were also decreased in cases 5 and 6 and 23, increased in cases 1 and 12, and normal in the others.

**Table 2:** Hormonal levels and family history of 25 patients with familial DSD

Case	Age at assays	Testosterone (ng/ml)		LH basal (IU/L)	FSH basal (IU/L)	INHIBIN B (pg/mL)	AMH (pmol/L)	Parent (family history)
		Before hCG	After hCG					
1	1 d	2.7		0.7^b^	<0.4^b^	178^b^	2348^b^	mother (premature menopause (27 years) in sister of his mother)
2	7 d	0.3		1.5	<0.4	186	794	mother (DSD in brother, sister and nephew)
3	3 d	0.2		<0.4	<0.4	>400	>600	mother (brother of case 2)
4	3 d	4	5.5	0.8^b^	1.3^b^	ND	614^b^	father (secondary infertility)
5^a^	3 d	0.1		0.1	0.4	<3	<1	father (infertility in 2 female cousins)
6	4 d	<0.07	<0.07	3.3	11	<10^b^	1^b^	father (premature menopause (38 years) in cousin)
7^a^	14 d	0.1	0.25	0.2	3.9	ND		mother (DSD in one nephew, congenital heart disease; brother with micropenis and cryptorchidism)
8^a^	23 d	0.2	0.9	0.3	1.8		78^b^	mother (premature menopause (28 years)
9	0.1 y	2		3.6	2.2	272	955	mother (hypospadias in each son of her 2 sisters)
10	0.1 y	2.1		3.2	2.4	176	630	father (cryptorchidism)
11	0.1 y	0.1		4.6	0.92	179	865	mother (brother of cases 2 and 3)
12	0.3 y	1.2		0.9	1	590	1674	father (hypospadias)
13	0.4 y	1		2.4	0.7	347	1129	mother (brother DSD, nephew hypospadias) father (unilateral cryptorchidism and 2 meatus urinarus in brothers)
14	5 y			0.7	1	14	1136	brother (hypospadias)
15	0.8 y	0.02		<0.4	0.6	175	1042	father (cryptorchidism)
16	0.8 y	<0.05		<0.4	<0.4	253	1245	mother (half brother of case 24)
17^a^	1.5 y	<0.05	<0.05	1.7	45	ND		mother (DSD in uncle)
18	1.8 y			<0.4	<0.4	94	485	father (hypospadias in brother)
19	3 y	<0.07		<0.2	0.47	77	1086	father (hypospadias in brother)
20	3.5 y	<0.07	3.3		ND			father (infertility in one sister and in paternal grandmother)
21	3.9 y				0.6	97	593	father (unilateral cryptorchidism as in cousin)
22	7.6 y				ND	44	719	brother (hypospadias)
23	10 y	1.1	1.6	<0.2	1.5	ND	37	brother (left cryptorchidism)
24	13.6 y	1.2		0.9	0.5	217	64	mother (half brother of case 16)
25	15 y	10.3		4.6	4.3	157	165	mother (uncle of cases 2, 3, 11)

^a^Genetic mutations (reference): case 5 SRY p.W98 C; case 7 GATA4 p.Gly221Arg (9); case 8 NR5A1 c.390delG (8); case 17 FOF2/ZFPM 2p.S402R (10)

Consanguinity reported in case 23

Cases 2,3,11 are brothers and case 25 is their maternal uncle. Cases 16 and 24 has the same mother (see pedigree)

^b^Age at assays similar except case 1 at 1.2 y, case 4 at 9 y, case 6 at 10 y, case 8 at 8.5 y

Müllerian structures were seen in the 4 cases with gonadal dysgenesis at ultrasound evaluation (cases 5 and 20), genitography (case 17) or MRI (case 6). This was confirmed at surgery in all cases, except in case 5, in which no operation had been performed. Surgery discovered right dysgerminoma in case 6 and right gonadoblastoma in case 20.

### Anorchia

No patient presented with somatic anomalies (Table 3). The mother of case 26 began menstruating at 12 years of age. She was operated on for scalenus syndrome. She had a previous medical pregnancy interruption at 20 weeks because of hygroma and anasarque. The female fetus had a 46,XX blood karyotype, intrauterine growth retardation, retrocervical edema, retrognathism, clinodactyly of the 5^th^ digit, agenesis of the 12^th^ pair of ribs, and ovaries with germ cells but no primary follicles. The mother of case 27 began menstruating at 9.5 years of age, then had irregular menstruation with increased basal plasma FSH concentrations and underwent 4 attempts at in vitro fertilizations before this pregnancy. After the proband was born, she underwent insemination, which led to an empty follicle. Her own mother experienced menopause at 50 years of age and had a hysterectomy for a fibroma. The father had a normal spermogram.

**Table 3:** Characteristics of 7 patients with familial form of anorchia

Case	Testes at birth		Age at first evaluation	Testosterone (ng/mL)	LH (IU/L)	FSH (IU/L)	Inhibin B (pg/mL)	AMH (pmol/L)	Parent (family history)
	Right	Left							
26	p	np	1d	ND	<0,4^b^	<0,4^b^	und	5	mother and fetus sister (see Results)
27	p	p	1.3y	und	5.5	68	und	2.5	mother (see Results)
28	torsion at birth	torsion at birth	3.2y	0.15	6^b^	34^b^	ND	ND	father (secondary infertility with oligoasthenospermia)
29	ND	ND	2y	und^a^	<0,2^b^	18^b^	ND	und	father (increased basal plasma FSH concentration (12 IU/L) at 38 y)
30	ND	ND	6.2y	und^a^	0.5	0.05	und	und	mother (cryptorchidism in cousin)
31	ND		8.2y	ND		2.8	und	und	father (cryptorchidism in son of sister)
32	np	np	13.6y	0.09	25	122	ND	ND	mother (premature menopause at 28 y)

*ND* not determined, *und* undetectable

^a^No increase in testosterone after hCG test

^b^Age at assays similar except case 26 at 0.8 y, 28 at 12.6, 29 at 6 y

At the first evaluation, all patients had unpalpable testes and normal penis length and morphology, except case 26, which presented with micropenis. The basal plasma concentrations of inhibin B, AMH and testosterone were very low or undetectable in all of the patients evaluated. The plasma concentrations of FSH were increased, except in cases 26, 30 and 31. Surgery showed no testicular tissue (cases 26 and 30) or unilateral or bilateral residues in the others.

### Modes of inheritance and genetic analyses

Figure 1 shows the pedigrees of the families. In addition to the genital anomalies, families reported premature menopause (cases 1, 6, 8, and 32) or infertility (cases 4, 5, 20, and 28). Assuming that DSD and infertility have the same genetic basis in these families, the mode of transmission of the phenotype in all families is suggestive of an autosomal dominant inheritance with a high degree of incomplete penetrance. Some pedigrees (eg index case 9 and index case 17) are indicative of a sex-limited expression. Consanguinity was present in case 23, in which the parents were first cousins. Mutations were identified in one patient with female external genitalia and 3/12 patients with genital ambiguity (25%) in the genes *SRY* (case 5), a heterozygous *NR5A1* mutation (case 8) [8], heterozygous *GATA4* (case 7) [9] and heterozygous *FOG2/ZFPM2* mutation (case 17) [10]. The inheritance of the *SRY* mutation is unknown because the father’s DNA was not available for study. However, he has two female cousins who were reported to be infertile. In each of the 3 other cases, the mother transmitted heterozygous mutation. The mother of case 8, who carries the *NR5A1* mutation, underwent premature menopause. The other mothers carrying mutations in *GATA4* or *FOG2* had apparently normal ovarian function (detailed in [9, 10]). In case 17, the heterozygous *FOG2/ZFPM2* mutation was carried by the unaffected mother and grandmother suggesting that mutations in this gene are associated with a sex-limited phenotype.

All other patients with DSD were sequenced for the *SRY, NR5A1, GATA4, FOG2, INSL3, LGR8 and MAP3K1* genes, and no pathogenic mutations were identified. All patients with anorchia were sequenced for *SRY, NR5A1, INSL3, MAMLD1* and the T222P variant of *LGR8* and no pathogenic mutations were identified.

## Discussion

We report, for the first time, a large monocentric study of familial forms of 46,XY DSD. This study shows a high frequency of the familial forms compared to the sporadic forms seen over 33 years by the same pediatric endocrinologist (22% of the DSD and 27% of the anorchia) when premature menopause or infertility are included.

### Biological data

The analysis of the biological data enables us 1) to define causes of DSD responsible for familial forms (androgen insensitivity, defects of testosterone synthesis, 5α-reductase deficiency); 2) to evaluate the Leydig and tubular functions; and 3) to compare this function in the same family and also its evolution with time if the individuals are of different ages. By this approach congenital adrenal hyperplasia or insufficiency as well as other causes of familial DSD were excluded. The plasma concentrations of testosterone (basal and after hCG test), indicating normal secretion associated with normal plasma luteinizing hormone (LH) concentrations during the first months, make the diagnoses of androgen insensitivity [16], defects of testosterone biosynthesis or Leydig cell hypoplasia less likely. Interestingly, the 7/13 of the cases evaluated during the first 6 months of life with low basal testosterone concentrations included the 4 in the series with mutation and 3 brothers (See below). In addition, mutations in exons 1 and 2–8 of the androgen receptor gene were excluded in 8 patients, including 1/2 with increased plasma AMH concentrations [19]. These concentrations, as well as those of inhibin B, provide information on the tubular function during childhood. They are undetectable in the patients with anorchia and in those with gonadal dysgenesis. Thus, in the patient carrying the *SRY* mutation, there was discordance between the 46,XY karyotype in utero and the absence of virilization of the external genitalia. The undetectable levels of testosterone and AMH in the amniotic fluid also excluded androgen insensitivity and suggested gonadal dysgenesis.

### Phenotypic variability

We and other investigators have observed variable expression of the clinical phenotypes as well as incomplete penetrance among family members carrying the same pathogenic mutation. Although rare, phenotypically normal brothers, fathers and paternal uncles of patients carrying the same *SRY* have been described [11]. None of these mutations has been found by population screening of large populations of unaffected 46,XY men. Many cases of DSD may be are familial rather than sporadic, as sometimes the effect on the phenotype can be so mild that the unsuspecting clinician may not diagnose mildly affected individuals until a more severely affected family members seeks medical attention. Baetens et al. [26] reported an *NR5A1* mutation in a proband with hypospadias and in his asymptomatic mother (regular menses at 30 years of age), maternal aunt (who had been diagnosed with primary ovarian insufficiency at 35), and maternal grandfather (who had been operated on for proximal hypospadias but spontaneously fathered the two girls). Camats et al. [27] suggested that the phenotypic variability may be a manifestation of the time-dependent deterioration of gonadal tissue and/or steroid secretion. In our study, one family included three brothers (cases 2, 3 and 11) who were evaluated in the neonatal period and had similar biological presentations, with normal plasma concentrations of inhibin B and AMH but low basal plasma testosterone concentrations. Their maternal uncle (case 25) also presented at 15 years of age with DSD and had normal adult concentrations of testosterone but developed azoospermia, despite initial normal inhibin B and AMH concentrations, suggesting testicular regression. Genomic sequencing may help to determine the source of this variability by revealing genetic modifiers that likely result from interactions between coding and regulatory variants resulting in an increased penetrance of rare pathogenic coding mutations [28].

We included patients with anorchia in this study because the familial occurrence of anorchia [2, 3, 5] and its association with other anomalies including gonadal dysgenesis suggest a genetic origin, but the genetic cause remains unknown, except in one case, which was reported to carry an *NR5A1* mutation [29]. The families of some patients with anorchia may include other individuals with pure or partial 46,XY gonadal dysgenesis. This has led to the hypothesis that anorchia is part of the clinical spectrum of 46,XY gonadal dysgenesis [7]. However, exploratory laparoscopy has suggested that at least some cases of anorchia are the result of prenatal testicular vascular accident associated with torsion during testicle descent [30].

We report a family history of reproductive problems in 6/25 (24%) patients with DSD and in 2/7 (29%) patients with anorchia. Two fathers are secondarily azoospermic and two mothers presented with premature menopause, suggesting a deterioration of their gonadal tissue over time. A link between gonadal development and infertility is becoming more evident as our understanding of the genes involved in gonad formation increases. For example, mutations in *NR5A1* cause DSD as well as male [31] and female infertility [8].

### Genetic analyses

A mutation associated with the phenotype was found in 4/14 patients presenting with ambiguous genitalia. Barseghyan et al. [32] recently found that reported data suggest that 15% of 46,XY gonadal dysgenesis cases are due to *SRY* mutations, 13% to mutations in *NR5A1*, 10–18% to *MAP3K1* variants, and a few cases to other rare genetic causes. The risk of malignancy is estimated to be approximately 30% in 46,XY individuals with DSD [33]. Among the 3 patients who underwent gonadectomies because of gonadal dysgenesis, two had gonadoblastoma and/or dysgerminoma, while the third operated on at 2 years of age had no tumor. This suggests that information to make an accurate diagnosis as well as information on the wider family members by the physician is of great importance. We did not perform this step, as it is sensitive in terms of the relationships and information sharing within the families.

### Study limitations

The present study has limitations because it is retrospective. No biological data of the affected members are available. The link between the DSD or anorchia of the proband and the symptoms in his/her family is probable but not confirmed, except in 75% of families carrying the mutation. Other causes of premature menopause or infertility were not excluded, as only one mother shares the *NR5A1* mutation with her son. We have no data on the infertility. Two fathers (of cases 4 and 28) were explored for secondary infertility, one of them has oligoasthernospermia. We retained cryptorchidism as a possible marker of familial form in 3 fathers (also present in a cousin in one case) and in one brother of the only patient of this series with consanguinity. Androgen insensitivity was excluded by the absence of mutation in only 8 patients, but the clinical-biological and evolutive (increase of the genital tubercle after testosterone administration) data make this diagnosis less likely. The exclusion of the diagnosis of 5α-reductase deficiency was based on the normal testosterone to dihydrotestosterone ratio, except in 3 cases where a mutation was excluded. There have been instances reported in the littérature of affected individuals who did not meet this hormonal criteria [34].

## Conclusions

This study is the first to demonstrate the high frequency of familial forms of 46,XY DSD and anorchia when premature menopause and male infertility are included. It also demonstrates the high variability of the expression of the phenotype within the families and the probable deterioration of fertility indicated by secondary sterility in two fathers and by premature menopause in two mothers. It poses the question of the risk of malignancy in the other affected members of the families, particularly in those with gonadal dysgenesis, as the collection of information within the families on this subject is difficult. The study also highlights the need to the physician to gather information on the fertility status of other members of the family. This could be important to identify familial cases, understand modes of transmission of the phenotype and eventually understand the genetic factors that are involved.

## Abbreviations

AMH: Anti-müllerian hormone
DSD: Disorders of sex development
FSH: Follicle stimulating hormone
hCG: Human chorionic gonadotropin
LH: Luteinising hormone
MRI: Magnetic resonance imaging
